# Supplementary material for: The Impact of Dietary Intake of Furocoumarins and Furocoumarin-Rich Foods on the Risk of Cutaneous Melanoma: A Systematic Review
Source: Nutrients. 2025 Apr 8;17(8):1296. doi: 10.3390/nu17081296 (PMC12030158; doi:10.3390/nu17081296)
Supplement: Supplementary file 1 [file nutrients-17-01296-s001.zip › nutrients-3528514-supplementary.pdf]

Table S1. Source publications used for forward snowballing during the literature search.

Publication
Melough MM, Kim K, Cho E, Chun OK. Relationship between Furocoumarin Intake and Melanoma History among US Adults in the National Health and Nutrition Examination Survey 2003-2012. Nutr Cancer. 2020;72(1):24-32.
Mahamat-Saleh Y, Cervenka I, Al-Rahmoun M, Mancini FR, Severi G, Ghiasvand R, et al. Citrus intake and risk of skin cancer in the European Prospective Investigation into Cancer and Nutrition cohort (EPIC). Eur J Epidemiol. 2020;35(11):1057-67.
Feskanich D, Willett WC, Hunter DJ, Colditz GA. Dietary intakes of vitamins A, C, and E and risk of melanoma in two cohorts of women. Br J Cancer. 2003;88(9):1381-7.
Malavolti M, Malagoli C, Fiorentini C, Longo C, Farnetani F, Ricci C, et al. Association between dietary vitamin C and risk of cutaneous melanoma in a population of Northern Italy. Int J Vitam Nutr Res. 2013;83(5):291-8.
Marley AR, Li M, Champion VL, Song Y, Han J, Li X. The association between citrus consumption and melanoma risk in the UK Biobank*. British Journal of Dermatology. 2021;185(2):353-62.
Sun WY, Rice MS, Park MK, Chun OK, Melough MM, Nan HM, et al. Intake of Furocoumarins and Risk of Skin Cancer in 2 Prospective US Cohort Studies. J Nutr. 2020;150(6):1535-44.
Melough MM, Wu SW, Li WQ, Eaton C, Nan HM, Snetselaar L, et al. Citrus Consumption and Risk of Cutaneous Malignant Melanoma in the Women's Health Initiative. Nutr Cancer. 2020;72(4):568-75.
Wu SW, Han JL, Feskanich D, Cho E, Stampfer MJ, Willett WC, et al. Citrus Consumption and Risk of Cutaneous Malignant Melanoma. J Clin Oncol. 2015;33(23):2500-U32.
Melough MM, Sakaki J, Liao LDM, Sinha R, Cho E, Chun OK. Association between Citrus Consumption and Melanoma Risk in the NIH-AARP Diet and Health Study. Nutr Cancer. 2021;73(9):1613-20.

Table S2. Search strings used for searching Pubmed, Scopus and Web of Science – Core Collection

**Pubmed**
((diet[Title/Abstract] OR nutrition[Title/Abstract] OR furocoumarin[Title/Abstract] OR citrus[Title/Abstract] OR "citrus fruits"[Title/Abstract] OR lemon[Title/Abstract] OR lime[Title/Abstract] OR grapefruit[Title/Abstract] OR "citrus sinensis"[Title/Abstract] OR "oranges"[Title/Abstract] OR tangerine[Title/Abstract] OR mandarin[Title/Abstract] OR clementine[Title/Abstract] OR pummelo[Title/Abstract] OR papeda[Title/Abstract] OR citron[Title/Abstract] OR "citrus juice"[Title/Abstract] OR "orange juice"[Title/Abstract] OR "lemon juice"[Title/Abstract] OR "lime juice"[Title/Abstract] OR "grapefruit juice"[Title/Abstract] OR "fig"[Title/Abstract] OR carrot[Title/Abstract] OR "carrot juice"[Title/Abstract] OR parsnip[Title/Abstract] OR turnip[Title/Abstract] OR celeriac[Title/Abstract] OR celery[Title/Abstract] OR parsley[Title/Abstract] OR dill[Title/Abstract] OR cilantro[Title/Abstract] OR cumin[Title/Abstract] OR coriander[Title/Abstract]) AND (melanoma[Title/Abstract] OR "malignant melanoma"[Title/Abstract]))<br>OR<br>((diet[Other Term] OR nutrition[Other Term] OR furocoumarin[Other Term] OR citrus[Other Term] OR "citrus fruits"[Other Term] OR lemon[Other Term] OR lime[Other Term] OR grapefruit[Other Term] OR "citrus sinensis"[Other Term] OR "oranges"[Other Term] OR tangerine[Other Term] OR mandarin[Other Term] OR clementine[Other Term] OR pummelo[Other Term] OR papeda[Other Term] OR citron[Other Term] OR "citrus juice"[Other Term] OR "orange juice"[Other Term] OR "lemon juice"[Other Term] OR "lime juice"[Other Term] OR "grapefruit juice"[Other Term] OR "fig"[Other Term] OR carrot[Other Term] OR "carrot juice"[Other Term] OR parsnip[Other Term] OR turnip[Other Term] OR celeriac[Other Term] OR celery[Other Term] OR parsley[Other Term] OR dill[Other Term] OR cilantro[Other Term] OR cumin[Other Term] OR coriander[Other Term]) AND (melanoma[Other Term] OR "malignant melanoma"[Other Term]))
**Scopus**
TITLE-ABS-KEY ((diet OR nutrition OR furocoumarin OR citrus OR "citrus fruits" OR lemon OR lime OR grapefruit OR "citrus sinensis" OR {oranges} OR tangerine OR mandarin OR clementine OR pummelo OR papeda OR citron OR "citrus juice" OR "orange juice" OR "lemon juice" OR "lime juice" OR "grapefruit juice" OR {fig} OR carrot OR "carrot juice" OR parsnip OR turnip OR celeriac OR celery OR parsley OR dill OR cilantro OR cumin OR coriander) AND (melanoma OR "malignant melanoma"))
**Web of Science – Core Collection**
TS=((diet OR nutrition OR furocoumarin OR citrus OR "citrus fruits" OR lemon OR lime OR grapefruit OR “citrus sinensis” OR “oranges” OR tangerine OR mandarin OR clementine OR pummelo OR papeda OR citron OR “citrus juice” OR “orange juice” OR “lemon juice” OR “lime juice” OR “grapefruit juice” OR “fig” OR carrot OR “carrot juice” OR parsnip OR turnip OR celeriac OR celery OR parsley OR dill OR cilantro OR cumin OR coriander) AND (melanoma OR "malignant melanoma"))

Table S3. References excluded during full-text screening and reasons for exclusion

**Reference**	**Reason for exclusion**
**References identified via database search**	
Bain, C.; Green, A.; Siskind, V.; Alexander, J.; Harvey, P. Diet and melanoma. An exploratory case-control study. *Ann Epidemiol* **1993**, *3*, 235-238, doi:10.1016/1047-2797(93)90024-x.	(1)
Berwick, M. Dietary advice for melanoma: Not ready for prime time. *Journal of Clinical Oncology* **2015**, *33*, 2487-2488, doi:10.1200/jco.2015.61.8116.	(2)
Berwick, M. Reply to S. Lehrer et al and J.C. Dowdy and R.M. Sayre. *Journal of Clinical Oncology* **2016**, *34*, 638, doi:10.1200/jco.2015.64.9384.	(2)
Bosshardt, O.P.; Grichnik, J.M. Citrus and melanoma risk: better to consume with dinner? *Dermatol Ther* **2016**, *29*, 211, doi:10.1111/dth.12294.	(2)
Brunk, D. Citrus consumption linked to risk of cutaneous malignant melanoma. *Oncology Report* **2015**, *11*, 12-13.	(2)
Burrill, G.S. A decade of change. *BioProcess International* **2012**, *10*, 62-64.	(1) & (2)
Chan, W. Nutritional aspects of the development of cancer. *Nutrition & Food Science* **2000**, *30*, 174-177, doi:10.1108/00346650010329399.	(2)
Cho, E.; Li, T.; Qureshi, A.A. Adolescent citrus fruit intake and risk of melanoma in US women. *Journal of Investigative Dermatology* **2016**, *136*, S107-S107, doi:10.1016/j.jid.2016.02.642.	(2)
DeWeerdt, S. The edible skincare diet. *Nature* **2018**, *563*, S94-S95, doi:10.1038/d41586-018-07433-7.	(2)
Dhana, A.; Yen, H.; Qureshi, A.A.; Cho, E. Epidemiology of Diet and Melanoma-Letter. *Cancer Epidemiol Biomarkers Prev* **2019**, *28*, 420, doi:10.1158/1055-9965.Epi-18-0882.	(2)
Dowdy, J.C.; Sayre, R.M. Melanoma risk from dietary furocoumarins: How much more evidence is required? *Journal of Clinical Oncology* **2016**, *34*, 636-637, doi:10.1200/jco.2015.63.8643.	(2)
Fan, Y.; Hu, C.; Xie, X.; Weng, Y.; Chen, C.; Wang, Z.; He, X.; Jiang, D.; Huang, S.; Hu, Z., et al. Effects of diets on risks of cancer and the mediating role of metabolites. *Nat. Commun.* **2024**, *15*, doi:10.1038/s41467-024-50258-4.	(1)
Fortes, C.; Mastroeni, S.; Melchi, F.; Pilla, M.A.; Antonelli, G.; Pasquini, P. Mediterranean diet and cutaneous melanoma. *Ann. Nutr. Metab.* **2007**, *51*, 91-91.	(2)
Fortes, C.; Mastroeni, S.; Melchi, F.; Pilla, M.A.; Antonelli, G.; Camaione, D.; Alotto, M.; Pasquini, P. A Protective Effect of the Mediterranean Diet for Cutaneous Melanoma. *Epidemiol. Prev.* **2010**, *34*, 60-60.	(2)
Fortes, C.; Mastroeni, S.; Levati, L.; Alotto, M.; Ricci, F.; D'Atri, S. The potentialimpact of dietary choices on melanoma risk: an anti-inflammatory diet. *Genes Nutr.* **2024**, *19*, 8, doi:10.1186/s12263-024-00745-6.	(1)
Gallagher, R.P.; Elwood, J.M.; Hill, G.B. Risk factors for cutaneous malignant melanoma: the Western Canada Melanoma Study. *Recent results in cancer research. Fortschritte der Krebsforschung. Progrès dans les recherches sur le cancer* **1986**, *102*, 38-55, doi:10.1007/978-3-642-82641-2_4.	(1)
Godar, D.E. Can dietary furocoumarins really be responsible for the increase in melanoma? *Medical Hypotheses* **2008**, *71*, 613-614, doi:10.1016/j.mehy.2008.05.008.	(2)
Grant-Kels, J.M. Nutrition and the skin: "You are what you eat" Part II. *Clinics in Dermatology* **2010**, *28*, 597, doi:10.1016/j.clindermatol.2010.03.035.	(1) & (2)
Hinds, M.W. Nonsolar factors in the etiology of malignant melanoma. *National Cancer Institute Monograph* **1982**, *Vol. 62*, 173-178.	(1)
Hogan, D.J. Diet and the Etiology of Melanoma. *N. Z. Med. J.* **1986**, *99*, 553-553.	(2)

Hohmann, C.B.; Bonamigo, R.R.; Segatto, M.M.; Costa, M.M.; Mastroeni, S.; Fortes, C. Could a specific dietary intake be a risk factor for cutaneous melanoma? *Cutis* **2016**, *97*, 421-425.	(1)
Hutchinson, E.; Novak, K.; McCarthy, N. In this issue. *Nature Reviews Cancer* **2005**, *5*, 911, doi:10.1038/nrc1774.	(1) & (2)
Katsimpris, A.; Antoniadis, A.G.; Dessypris, N.; Karampinos, K.; Gogas, H.J.; Petridou, E.T. Associations of Dietary Factors with Cutaneous Melanoma: A Case-Control Study in Greece with Literature Review. *Oncol. Res. Treat.* **2024**, *47*, 206-217, doi:10.1159/000538278.	(1)
Kirkpatrick, C.S.; White, E.; Lee, J.A.H. Case-Control Study of Malignant-Melanoma in Washington-State .2. Diet, Alcohol, and Obesity. *American Journal of Epidemiology* **1994**, *139*, 869-880, doi:10.1093/oxfordjournals.aje.a117093.	(1)
Lehrer, S.; Green, S.; Rosenzweig, K.E. Melanoma Risk and Citrus Consumption. *J Clin Oncol* **2016**, *34*, 636, doi:10.1200/jco.2015.63.8254.	(2)
Le Marchand, L.; Saltzman, B.S.; Hankin, J.H.; Wilkens, L.R.; Franke, A.A.; Morris, S.J.; Kolonel, L.N. Sun exposure, diet, and melanoma in Hawaii Caucasians. *Am J Epidemiol* **2006**, *164*, 232-245, doi:10.1093/aje/kwj115.	(1)
Mackie, B.S.; Mackie, L.E. Prevention of Melanoma. *Nutrition and Cancer* **1990**, *14*, doi:10.1080/01635589009514080.	(2)
Mahamat-Saleh, Y. Is consumption of citrus associated with a higher risk of melanoma? *Br J Dermatol* **2021**, *185*, 245-246, doi:10.1111/bjd.20408.	(2)
Mahamat-Saleh, Y.; Cervenka, I.; Al Rahmoun, M.; Savoye, I.; Mancini, F.R.; Trichopoulou, A.; Boutron-Ruault, M.C.; Kvaskoff, M. Mediterranean dietary pattern and skin cancer risk: A prospective cohort study in French women. *Am J Clin Nutr* **2019**, *110*, 993-1002, doi:10.1093/ajcn/nqz173.	(1)
Malagoli, C.; Malavolti, M.; Agnoli, C.; Crespi, C.M.; Fiorentini, C.; Farnetani, F.; Longo, C.; Ricci, C.; Albertini, G.; Lanzoni, A., et al. Diet Quality and Risk of Melanoma in an Italian Population. *J Nutr* **2015**, *145*, 1800-1807, doi:10.3945/jn.114.209320.	(1)
Marrelli, M.; Statti, G.A.; Tundis, R.; Menichini, F.; Conforti, F. Fatty acids, coumarins and polyphenolic compounds of Ficus carica L. cv. Dottato: Variation of bioactive compounds and biological activity of aerial parts. *Natural Product Research* **2014**, *28*, 271-274, doi:10.1080/14786419.2013.841689.	(1)
Martens, M.C.; Seebode, C.; Lehmann, J.; Emmert, S. Molecular Mechanisms of Cutaneous Photocarcinogenesis: An Update. *Aktuelle Dermatologie* **2018**, *44*, 226-231, doi:10.1055/s-0043-122350.	(2)
McGovern, V. Save our (young) skins! *Environmental Health Perspectives* **2005**, *113*.	(2)
Meyskens Jr, F.L.; Farmer, P.J.; Anton-Culver, H. Diet and melanoma in a case-control study. *Cancer Epidemiology Biomarkers and Prevention* **2005**, *14*, 293.	(2)
Meyskens, F.L., Jr.; Farmer, P.J.; Anton-Culver, H. Diet and melanoma in a case-control study. *Cancer Epidemiol Biomarkers Prev* **2005**, *14*, 293.	(3)
Millen, A.; Tucker, M.A.; Hartge, P.; Potischman, N. Diet and risk of malignant melanoma in a case-control study. *Cancer Epidemiol. Biomarkers Prev.* **2003**, *12*, 1315S-1315S.	(2)
Murzaku, E.C.; Bronsnick, T.; Rao, B.K. Diet in dermatology. Part II. Melanoma, chronic urticaria, and psoriasis (vol 71, pg 1053, 2014). *Journal of the American Academy of Dermatology* **2015**, *73*, 353-353.	(1)
Murzaku, E.C.; Bronsnick, T.; Rao, B.K. Erratum: Diet in dermatology. Part II. Melanoma, chronic urticaria, and psoriasis (Journal of the American Academy of Dermatology (2014) 71 (1053-1068)). *Journal of the American Academy of Dermatology* **2015**, *73*, 353, doi:10.1016/j.jaad.2015.05.036.	(2)
Navarro-Bielsa, A.; Gracia-Cazaña, T.; Almagro, M.; De-la-Fuente-Meira, S.; Florez, Á.; Yélamos, O.; Montero-Vilchez, T.; González-Cruz, C.; Diago, A.; Abadías-Granado, I., et al.	(4)

Multicenter, Prospective, Case-control Study of Exposome in Melanoma. *Actas Dermosifiliogr* **2025**, 10.1016/j.ad.2024.09.022, doi:10.1016/j.ad.2024.09.022.	
Ossterlind, A. Malignant melanoma in Denmark occurrence and risk factors. *Acta Oncologica* **1990**, *29*, 833-854, doi:10.3109/02841869009096378.	(1)
Parent, M.E.; El-Zein, M.; Rousseau, M.C.; Pintos, J.; Siemiatycki, J. The authors reply. *American Journal of Epidemiology* **2013**, *177*, 1166-1167, doi:10.1093/aje/kwt074.	(1) & (2)
Preventing cancer. *Nature* **2006**, *442*, 720, doi:10.1038/442720a.	(1) & (2)
Shang, X.; Liu, J.; Zhu, Z.; Zhang, X.; Huang, Y.; Liu, S.; Wang, W.; Zhang, X.; Tang, S.; Hu, Y., et al. Healthy dietary patterns and the risk of individual chronic diseases in community-dwelling adults. *Nat. Commun.* **2023**, *14*, doi:10.1038/s41467-023-42523-9.	(1)
Shors, A.R.; Solomon, C.; McTiernan, A.; White, E. Melanoma risk in relation to height, weight, and exercise (United States). *Cancer Causes Control* **2001**, *12*, 599-606, doi:10.1023/a:1011211615524.	(1)
Soliman, A.; Moon, T.; Ritenbaugh, C.K. Relationship of Diet and Melanoma in Arizona. *Faseb J.* **1993**, *7*, A79-A79.	(2)
Stryker, W.S.; Stampfer, M.J.; Stein, E.A.; Kaplan, L.; Louis, T.A.; Sober, A.; Willett, W.C. Diet, plasma levels of beta-carotene and alpha-tocopherol, and risk of malignant melanoma. *Am J Epidemiol* **1990**, *131*, 597-611, doi:10.1093/oxfordjournals.aje.a115544.	(1)
Thesen, R.; Citrus fruits: Consumption seems to increase the risk of melanoma. *Pharmazeutische Zeitung* **2015**, *160*.	(2)
Vinceti, M.; Pellacani, G.; Malagoli, C.; Bassissi, S.; Sieri, S.; Bonvicini, F.; Krogh, V.; Seidenari, S. A population-based case-control study of diet and melanoma risk in northern Italy. *Public Health Nutr* **2005**, *8*, 1307-1314, doi:10.1079/phn2005754.	(1)
Wang, Q.; Qiu, Z.; Cheng, L.; Xu, S.; Li, H.; Guo, J.; Zhang, X. Is diet related to skin condition? A Mendelian randomization study. *Arch. Dermatol. Res.* **2024**, *316*, doi:10.1007/s00403-024-03103-z.	(1)
Weinstock, M.A.; Stampfer, M.J.; Lew, R.A.; Willett, W.C.; Sober, A.J. Case-control study of melanoma and dietary vitamin D: Implications for advocacy of sun protection and sunscreen use. *Journal of Investigative Dermatology* **1992**, *98*, 809-811, doi:10.1111/1523-1747.ep12499962.	(1)
Whiteman, D.C.; Webb, P.M.; Green, A.C.; Neale, R.E.; Fritschi, L.; Bain, C.J.; Parkin, D.M.; Wilson, L.F.; Olsen, C.M.; Nagle, C.M., et al. Cancers in Australia in 2010 attributable to modifiable factors: Summary and conclusions. *Australian and New Zealand Journal of Public Health* **2015**, *39*, 477-484, doi:10.1111/1753-6405.12471.	(1)
Wolf, R.; Parish, L.C. Controversies in dermatology: Part V. *Clinics in Dermatology* **2013**, *31*, 665, doi:10.1016/j.clindermatol.2013.05.019.	(1) & (2)
Wu, S.; Han, J.; Feskanich, D.; Stampfer, M.J.; Willett, W.C.; Qureshi, A.A. Citrus consumption is associated with risk of malignant melanoma. *Journal of Investigative Dermatology* **2014**, *134*, S59-S59.	(2)
Wu, S.; Cho, E.; Qureshi, A.A. Reply to S. Lehrer et al and J.C. Dowdy and R.M. Sayre. *Journal of Clinical Oncology* **2016**, *34*, 637-638, doi:10.1200/jco.2015.64.9376.	(2)
Yaghjyan, L.; Wijayabahu, A.T.; Egan, K.M. RE: The association between dietary quality and overall and cancer-specific mortality among cancer survivors, NHANES III. *JNCI Cancer Spectrum* **2018**, *2*, doi:10.1093/jncics/pky044.	(1) & (2)
Zaridze, D.; Mukeria, A.; Duffy, S.W. Risk factors for skin melanoma in Moscow. *International Journal of Cancer* **1992**, *52*, 159-161, doi:10.1002/ijc.2910520128.	(1)
**References identified via forward snowballing**	
Asgari, M.M.; Maruti, S.S.; Kushi, L.H.; White, E. Antioxidant supplementation and risk of incident melanomas: Results of a large prospective cohort study. Arch. Dermatol. 2009, 145, 879-882, doi:10.1001/archdermatol.2009.176.	(1)

Cheng, W.W.; Wang, Z.K.; Shangguan, H.F.; Zhu, Q.; Zhang, H.Y. Are vitamins relevant to cancer risks? A Mendelian randomization investigation. Nutrition 2020, 78, doi:10.1016/j.nut.2020.110870.	(1)
Filippini, T.; Tancredi, S.; Malagoli, C.; Malavolti, M.; Bargellini, A.; Vescovi, L.; Nicolini, F.; Vinceti, M. Dietary Estimated Intake of Trace Elements: Risk Assessment in an Italian Population. Expos. Health 2020, 12, 641-655, doi:10.1007/s12403-019-00324-w.	(1)
Melough, M.M.; Sakaki, J.; Liao, L.M.; Sinha, R.; Cho, E.; Chun, O.K. Association between Citrus Consumption and Melanoma Risk in the NIH-AARP Diet and Health Study. Nutrition and Cancer 2021, 73, 1613-1620, doi:10.1080/01635581.2020.1803933.	(3)
Sable, K.A.; Shields, B.E. The Role of Dietary Antioxidants in Melanoma and Nonmelanoma Skin Cancer. Cutis 2023, 111, 33-38, doi:10.12788/cutis.0672.	(2)
Wang, K.; Jiang, H.; Li, W.; Qiang, M.; Dong, T.; Li, H. Role of vitamin C in skin diseases. Front. Physiol. 2018, 9, doi:10.3389/fphys.2018.00819.	(2)

(1)= wrong topic with no information on CM risk of furocoumarin-containing food, (2)= wrong publication type, (3)= duplicate, (4)= wrong language

Table S4. Categories for exposure measurement and reporting by publication and food group.

Publication	Categories used for exposure measurement	Categories used for exposure reporting
**Citrus fruits and juices**		
Mahamat-Saleh [35]	grams/day	quartiles
Marley [38]	Fruits: quantity per day (1/2,1,2,3,4+) Juices: number of glasses (250ml) per day (1/2, 1,2,3,4,5,6+)	portions/day (5 categories)
Melough [37]	9 frequency options from never to 2+ servings per day, serving of fruit: 1 orange or 1⁄2 grapefruit, serving of juice: 177.5 ml glass	portions/day or week (5 categories)
Melough [39]	Juices: 10 frequency options from never to 6+ times per day, with 3 serving options from less than 3⁄4 cup to more than 1 cup. Fruits: 10 frequency options from never to 2+ times per day, with 3 serving options from less than 1 serving to more than 1 serving. 1 serving of fruit: 1 orange or 1⁄2 grapefruit respectively.	cups/day or week (5 categories)
Millen [31]	0 to 3/day in the previous year. No unit described.	consumption times/day (4 categories)
Wu [26]	6 frequency options from never to 6 servings per day (average over previous year), serving of fruit: 1 orange or 1⁄2 grapefruit, serving of juice: 177.5 ml glass	portions/day or week (5 categories)
**Citrus fruits**		
Fortes [32]	7 frequency options: "never", "less than monthly", "less than weekly", "one to two times per week", "three to four times per week", "five to seven times per week", "daily".	consumption times/week (3 categories)
Mahamat-Saleh [35]	grams/day	quartiles
Malagoli [23]	grams/day	tertiles
Melough [37]	9 frequency options from never to 2+ servings per day, serving of fruit: 1 orange or 1⁄2 grapefruit	portions/week (5 categories)
Melough [39]	Fruits: 10 frequency options from never to 2+ times per day, with 3 serving options from less than 1 serving to more than 1 serving. 1 serving of fruit: 1 orange or 1⁄2 grapefruit respectively.	cups/week (5 categories)
Vinceti [33]	3 portion options (“small”, “medium”, “large”) per day assessed by selecting a picture of the respective portion size	increment of 10g consumption/day (continuous variable)
**Citrus juices**		
Mahamat-Saleh [35]	grams/day	quartiles

Melough [37]	9 frequency options from never to 2+ servings per day, serving of juice: 177.5 ml glass	portions/day or week (5 categories)
Melough [39]	10 frequency options from never to 6+ times per day, with 3 serving options from less than 3⁄4 cup to more than 1 cup.	cups/week (5 categories)
**Grapefruit and grapefruit juice**		
**Grapefruit**		
Marley [38]	quantity per day (1/2,1,2,3,4+)	portions/day (5 categories)
Melough [39]	10 frequency options from never to 2+ times per day, with 3 serving options from less than 1 serving to more than 1 serving. 1 serving of fruit: 1 orange or 1⁄2 grapefruit respectively.	cups/week (5 categories)
Soliman [21]	Number of medium-sized servings per week. Medium-sized was not defined.	consumption times/week (3 categories)
Wu [26]	6 frequency options from never to 6 servings per day (average over previous year), serving of fruit: 1 orange or 1⁄2 grapefruit	portions/week (5 categories)
**Grapefruit juice**		
Marley [38]	number of glasses (250ml) per day (1/2, 1,2,3,4,5,6+)	portions/day (5 categories)
Wu [26]	6 frequency options from never to 6 servings per day (average over previous year), serving of juice: 177.5 ml glass	portions/week (5 categories)
**Oranges and orange juice**		
**Orange**		
Marley [38]	quantity of fruit per day (1/2,1,2,3,4+)	portions/day (5 categories)
Soliman [21]	Number of medium-sized servings per week. Medium-sized was not defined.	consumption times/week (3 categories)
Veierød [29]	not reported	no quantitative data reported
Wu [26]	6 frequency options from never to 6 servings per day (average over previous year), serving of fruit: 1 orange or 1⁄2 grapefruit	portions/week (5 categories)
**Orange juice**		
Feskanich [24]	6 frequency options from never to 6 servings per day (average over previous year), serving of juice: 177.5 ml glass	portions/week or day (5 categories)
Marley [38]	number of glasses (250ml) per day (1/2, 1,2,3,4,5,6+)	portions/day (5 categories)
Soliman [21]	Number of medium-sized servings per week. Medium-sized was not defined.	consumption times/week (3 categories)
Vinceti [33]	grams/day	tertiles
Wu [26]	6 frequency options from never to 6 servings per day (average over previous year), serving of juice: 177.5 ml glass	portions/week (5 categories)

**Other citrus fruits**		
**Orange, tangerine, tangelo**		
Melough [39]	10 frequency options from never to 2+ times per day, with 3 serving options from less than 1 serving to more than 1 serving. 1 serving of fruit: 1 orange or 1⁄2 grapefruit respectively.	cups/week (5 categories)
**Mandarin**		
Marley [38]	quantity of fruit per day (1/2,1,2,3,4+)	portions/day (4 categories)
**Orange and grapefruit**		
Malavolti [22]	grams/day	Tertiles (of vitamin C intake)
**Orange and mandarin**		
Grasgruber [34]	gram/day	gram/day
**Orange juice and grapefruit juice**		
Malavolti [22]	grams/day	Tertiles (of vitamin C intake)
**Tangerine**		
Malavolti [22]	grams/day	Tertiles (of vitamin C intake)
**Others**		
**Parsely**		
Fortes [32]	7 frequency options: "never", "less than monthly", "less than weekly", "one to two times per week", "three to four times per week", "five to seven times per week", "daily".	consumption/week (2 categories)
**Carrot**		
Fortes [32]	7 frequency options: "never", "less than monthly", "less than weekly", "one to two times per week", "three to four times per week", "five to seven times per week", "daily".	consumption/week (2 categories)
Holman [20]	not reported	no quantitative data reported
Naldi [30]	3 frequency options per week from low (0 servings/week), and medium (<1 serving/week) to high (≥1 serving per week), serving size not defined.	portions/week (3 categories)
Østerlind [27]	2 categories: "exposed" = carrot consumption ≥ 1 time/ month vs. "non-exposed" = carrot consumption < 1 time/ month	exposed vs. non-exposed (2 categories)
Soliman [21]	Number of medium-sized servings per week. Medium-sized was not defined.	consumption times/week (3 categories)
Stryker [28]	3 frequency options as consumption times per week (low, medium and high = at least 1 cup/week)	consumption/week (3 categories)
**Total furocoumarin consumption**		
Melough [36]	Furocoumarin consumption calculated by summing the furocoumarin content contributed by each food recorded in two 24-hour dietary recall interviews	percentiles (4 categories)

Sun [25]	average intake of foods in the previous year measured in 9 frequency options from “never” to “more than 6 servings per day” matched with furocoumarin database (accumulated)	Quintiles

Table S5. Detailed results of the individual studies and assigned type of association

Publication	Reported results				Type of association
**Citrus fruits and juices**					
	**Quartiles of intake**	**HR (95%-CI)**	**Adjusted for**	**p-trend**	
Mahamat-Saleh [35]	Q1	1.00 (ref)	age, education, BMI, smoking, alcohol intake, physical activity, and energy intake, total vegetable intake, coffee intake, non-citrus fruit intake, and non-citrus juice intake	0.96	o
	Q2	0.97 (0.83-1.13)			
	Q3	1.03 (0.88-1.21)			
	Q4	0.98 (0.83-1.15)			
	**Categories of intake**	**OR (95%-CI)**	**Adjusted for**	**p-trend**	
Marley [38]	None	1.0 (ref)	age, sex, education, income, physical activity, BMI, smoking status, alcohol intake, energy intake, coffee intake, tanning ability, childhood sunburn occasions, natural hair color, skin color, average time spent outdoors in summer, sunlamp or solarium use, use of sun/ultraviolet protection and history of NMSC	0.0051	+
	>0-0.5 portions/day	0.98 (0.82-1.16)			
	>0.5-1/day	1.07 (0.91-1.25)			
	>1-2 portions/day	1.13 (0.93-1.36)			
	>2 portions/day	1.63 (1.24-2.12)			
	**Categories of intake**	**HR (95%-CI)**	**Adjusted for**	**p-trend**	
Melough [37]	<2 portions/week	1.00 (ref)	age, BMI, education, physical activity, alcohol consumption, history of NMSC, regional solar radiation, skin reaction to sun, average daily time outdoors currently, sunscreen use	0.45	+
	2-4 portions/week	1.14 (0.95-1.37)			
	5-6 portions/week	0.90 (0.70-1.16)			
	1-1.4 portions/day	1.06 (0.88-1.28)			
	1.5+ portions/day	1.12 (0.91-1.37)			
	**Categories of intake**	**HR (95%-CI)**	**Adjusted for**	**p-trend**	
Melough [39]	None	1.00 (ref)	age, sex, cigarette smoking, BMI, education, alcohol intake, physical activity, family history of cancer, July erythemal exposure	0.41	o
	<1 cups/week	0.98 (0.77-1.25)			
	1 - <3.5 cups/week	1.15 (0.91-1.46)			
	0.5 - <1 cups/day	1.16 (0.92-1.47)			
	1+ cups/day	1.09 (0.86-1.39)			

Millen [31]	**Frequency of intake**	**OR**	**Adjusted for**	**p-trend**	-
	0-0.1 times/day	1.0 (ref)	age, sex, study site, confirmed dysplastic nevi status, education, and skin response after repeated/prolonged sun	0.19	
	0.2-0.5 times/day	0.75			
	0.6-1.0 times/day	0.59			
	1.1-3.0 times/day	0.77			
Wu [26]	**Categories of intake**	**HR (95%-CI)**	**Adjusted for**	**p-trend**	+
	<2 portions/week	1.0 (ref)	age, family history of melanoma, natural hair color, No. of arm moles, sunburn susceptibility as child or adolescent, No. of lifetime blistering sunburns, cumulative ultraviolet flux since baseline, average time spent in direct sunlight since high school, BMI, physical activity, smoking status, intake of total energy, alcohol, coffee, vitamin C from supplements, menopausal status and postmenopausal hormone use (only women)	<0.001	
	2-4 portions/week	1.10 (0.94-1.30)			
	5-6 portions/week	1.26 (1.08-1.47)			
	1-1.5 portions/day	1.27 (1.09-1.49)			
	≥1.6 portions/day	1.36 (1.14-1.63)			
**Citrus fruits**					
Fortes [32]	**Frequency of intake**	**OR (95%-CI)**	**Adjusted for**	**p-trend**	-
	Low (up to 2 times/week)	1.0 (ref)	age, sex, education, hair color, skin phototypes, number of nevi, presence of freckles in childhood and sunburns in childhood.	0.003	
	Medium (3-4 times/week)	0.79 (0.47-1.32)			
	High (>5 times/week)	0.51 (0.32-0.80)			
Mahamat-Saleh [35]	**Quartiles of intake**	**HR (95%-CI)**	**Adjusted for**	**p-trend**	+
	Q1	1.00 (ref)	age, education, BMI, smoking, alcohol intake, physical activity, energy intake, total vegetable intake, non-citrus fruit intake, citrus juice, and coffee intake	0.01	
	Q2	1.05 (0.90-1.22)			
	Q3	1.17 (0.99-1.37)			
	Q4	1.23 (1.02-1.48)			

Malagoli [23]	**Tertiles of daily intake**	**OR**	**Adjusted for**		o
	T1	1.0 (ref)	phototype, sunburn history, education, BMI, non-alcohol energy, vitamin C and vitamin D intake, Greek Mediterranean index and glycemic index		
	T2	0.97			
	T3	0.93			
	**Daily intake as continuous variable:**	1.01 (0.97-1.04)			
Melough [37]	**Categories of intake**	**HR (95%-CI)**	**Adjusted for**	**p-trend**	o
	Never	1.00 (ref)	age, BMI, education, physical activity, alcohol consumption, history of NMSC, regional solar radiation, skin reaction to sun, average daily time outdoors currently, sunscreen use	0.44	
	<1 portions/week	1.05 (0.86-1.28)			
	1 portion/week	1.03 (0.81-1.31)			
	2-4 portions/week	0.97 (0.76-1.23)			
	5+ portions/week	0.95 (0.76-1.20)			
Melough [39]	**Categories of intake**	**HR (95%-CI)**	**Adjusted for**	**p-trend**	o
	None	1.00 (ref)	age, sex, cigarette smoking, BMI, education, alcohol intake, physical activity, family history of cancer, July erythemal exposure	0.36	
	<0.25 cups/week	1.05 (0.91-1.21)			
	0.25 - <0.75 cups/week	1.07 (0.93-1.23)			
	0.75 - <2 cups/week	1.05 (0.91-1.22)			
	2+ cups/week	1.07 (0.93-1.23)			
Vinceti [33]	**Daily intake as continuous variable (per 10-g increments)**	**RR (95%-CI)** 1.05 (0.96-1.15)	No adjustment	**p-trend** 0.308	+
**Citrus juices**					
Mahamat-Saleh [35]	**Quartiles of intake**	**HR (95%-CI)**	**Adjusted for**	**p-trend**	o
	Q1	1.00 (ref)	age, education, BMI, smoking, alcohol intake, physical activity, energy intake, total vegetable intake, non-citrus juice, citrus fruit, and coffee intake	0.31	
	Q2	1.01 (0.84-1.21)			
	Q3	1.03 (0.87-1.22)			
	Q4	0.92 (0.78-1.08)			

Melough [37]	**Categories of intake**	**HR (95%-CI)**	**Adjusted for**	**p-trend**	+
	<1 portions/week	1.00 (ref)	age, BMI, education, physical activity, alcohol consumption, history of NMSC, regional solar radiation, skin reaction to sun, average daily time outdoors currently, sunscreen use	0.11	
	1 portion/week	0.86 (0.67-1.11)			
	2-4 portions/week	0.95 (0.78-1.15)			
	5-6 portions/week	1.08 (0.82-1.42)			
	1+ portions/day	1.13 (0.96-1.32)			
Melough [39]	**Categories of intake**	**HR (95%-CI)**	**Adjusted for**	**p-trend**	o
	None	1.00 (ref)	age, sex, cigarette smoking, BMI, education, alcohol intake, physical activity, family history of cancer, July erythemal exposure	0.9	
	<0.25 cups/week	1.06 (0.93-1.20)			
	0.25 - <0.5 cups/week	1.20 (1.04-1.39)			
	0.5 - <1 cups/week	1.14 (1.00-1.30)			
	1+ cups/week	1.02 (0.87-1.19)			
**Grapefruit and grapefruit juice**					
**Grapefruit**					
Marley [38]	**Categories of intake**	**OR (95%-CI)**	**Adjusted for**	**p-trend**	+
	None	1.0 (ref)	age, sex, education, income, physical activity, BMI, smoking status, alcohol intake, coffee intake, tanning ability, childhood sunburn occasions, natural hair color, skin color, average time spent outdoors in summer, sunlamp or solarium use, use of sun/ultraviolet protection, total energy intake, history of NMSC, consumption of other individual citrus products	0.089	
	>0-0.5 portions/day	1.04 (0.77-1.37)			
	>0.5-1 portions/day	1.54 (0.89-2.45)			
	>1 portions/day	2.41 (0.14-11.25)			
Melough [39]	**Categories of intake**	**HR (95%-CI)**	**Adjusted for**	**p-trend**	+
	None	1.00 (ref)	age, sex, cigarette smoking, BMI, education, alcohol intake, physical activity, family history of cancer, July erythemal exposure	0.5	
	<1 cups/week	1.10 (1.02-1.19)			
	1 - <2 cups/week	1.15 (0.94-1.41)			
	2 - <3 cups/week	1.21 (1.04-1.41)			
	3+ cups/week	1.10 (0.96-1.26)			

Soliman [21]	**Level of intake**	**OR**		**p-trend**	o
	Low	1.0 (ref)	No adjustment		
	Moderate	0.9		0.94	
	High	1.0			
Wu [26]	**Categories of intake**	**HR (95%-CI)**	**Adjusted for**	**p-trend**	+
	Never	1.0 (ref)	age, family history of melanoma, natural hair color, No. of arm moles, sunburn susceptibility as child or adolescent, No. of lifetime blistering sunburns, cumulative ultraviolet flux since baseline, average time spent in direct sunlight since high school, BMI, physical activity, smoking status, intake of total energy, alcohol, coffee, vitamin C supplements, menopausal status and postmenopausal hormone use (only women)	<0.001	
	<1 portions/week	1.17 (1.02-1.36)			
	1 portion/week	1.30 (1.10-1.53)			
	2 portions/week	1.33 (1.11-1.59)			
	≥3 portions/week	1.41 (1.10-1.82)			
**Grapefruit juice**					
Marley [38]	**Categories of intake**	**OR (95%-CI)**	**Adjusted for**	**p-trend**	+
	None	1.0 (ref)	age, sex, education, income, physical activity, BMI, smoking status, alcohol intake, coffee intake, tanning ability, childhood sunburn occasions, natural hair color, skin color, average time spent outdoors in summer, sunlamp or solarium use, use of sun/ultraviolet protection, total energy intake, history of NMSC, consumption of other individual citrus products	0.6	
	>0-0.5 portions/day	0.71 (0.46-1.03)			
	>0.5-1 portions/day	1.20 (0.71-1.89)			
	>1 portions/day	1.96 (0.48-5.23)			
Wu [26]	**Categories of intake**	**OR (95%-CI)**	**Adjusted for**	**p-trend**	o
	Never	1.0 (ref)	age, family history of melanoma, natural hair color, No. of arm moles, sunburn susceptibility as child or adolescent, No. of lifetime blistering sunburns, cumulative ultraviolet flux since baseline, average time spent in direct sunlight since high school, BMI, physical activity, smoking status, intake of total energy, alcohol, coffee, vitamin C supplements, menopausal status and postmenopausal hormone use (only women)	0.79	
	<1 portions/week	0.98 (0.87-1.10)			
	1 portion/week	0.98 (0.82-1.16)			
	2 portions/week	0.99 (0.81-1.20)			
	≥3 portions/week	0.98 (0.81-1.19)			

Oranges and orange juice					
**Orange**					
	**Categories of intake**	**OR (95%-CI)**	**Adjusted for**	**p-trend**	
Marley [38]	None	1.0 (ref)	age, sex, education, income, physical activity, BMI, smoking status, alcohol intake, coffee intake, tanning ability, childhood sunburn occasions, natural hair color, skin color, average time spent outdoors in summer, sunlamp or solarium use, use of sun/ultraviolet protection, total energy intake, history of NMSC, consumption of other individual citrus products	0.043	+
	>0-0.5 portions/day	1.00 (0.82-1.21)			
	>0.5-1 portions/day	1.11 (0.88-1.38)			
	>1 portions/day	1.79 (1.07-2.78)			
	**Level of intake**	**OR**	**Adjusted for**	**p-trend**	
Soliman [21]	Low	1.0 (ref)	age, sex, tanning, number of palpable moles, total calories, sun exposure, year of recruitment into the study	0.52	o
	Moderate	1.3			
	High	1.2			
Veierød [29]	No quantitative results: "The consumption of […] oranges […] were not associated with the incidence of CMM, both for men and women."				o
	**Categories of intake**	**HR (95%-CI)**	**Adjusted for**	**p-trend**	
Wu [26]	Never	1.0 (ref)	age, family history of melanoma, natural hair color, No. of arm moles, sunburn susceptibility as child or adolescent, No. of lifetime blistering sunburns, cumulative ultraviolet flux since baseline, average time spent in direct sunlight since high school, BMI, physical activity, smoking status, intake of total energy, alcohol, coffee, vitamin C supplements, menopausal status and postmenopausal hormone use (only women)	0.81	o
	≤1 portions/week	1.06 (0.88-1.28)			
	2 portions/week	0.97 (0.71-1.23)			
	3 portions/week	1.05 (0.85-1.31)			
	≥4 portions/week	1.08 (0.86-1.35)			
**Orange juice**					
	**Categories of intake**	**RR (95%-CI)**	**Adjusted for**	**p-trend**	
Feskanich [24]	Never	1.0 (ref)	age, follow-up cycle, skin reaction after 2h of sun exposure during childhood, number of sunburns, number of moles, hair color, family history of melanoma, state of residence, menopausal status, oral contraceptive use, postmenopausal hormone use, parity, height, BMI, total energy intake, multivitamin use, use of vitamin C, vitamin E, b-carotene supplements	0.008	+
	1-3 portions/month	1.09 (0.73-1.62)			
	1 portions/month	1.31 (0.83-2.05)			
	2-6 portions/week	1.44 (1.00-2.08)			
	≥1 portions/day	1.61 (0.92-2.84)			

Marley [38]	**Categories of intake**	**OR (95%-CI)**	**Adjusted for**	**p-trend**	+
	None	1.0 (ref)	age, sex, education, income, physical activity, BMI, smoking status, alcohol intake, coffee intake, tanning ability, childhood sunburn occasions, natural hair color, skin color, average time spent outdoors in summer, sunlamp or solarium use, use of sun/ultraviolet protection, total energy intake, history of NMSC, consumption of other individual citrus products	0.021	
	>0-0.5 portions/day	0.96 (0.82-1.13)			
	>0.5-1 portions/day	1.09 (0.92-1.29)			
	>1 portions/day	1.54 (1.10-2.10)			
Soliman [21]	**Categories of intake**	**OR**	**Adjusted for**	**p-trend**	o
	Low	1.0 (ref)	age, sex, tanning, number of palpable moles, total calories, sun exposure, year of recruitment into the study	0.96	
	Moderate	0.6			
	High	1.0			
Vinceti [33]	**Tertiles of intake**	**RR (95%-CI)**	**Adjusted for**	**p-trend**	o
	T1	1.0 (ref)	total energy intake, family history of melanoma, skin type, history of sunlight exposure and sunburns	0.518	
	T2	1.81 (0.66-4.97)			
	T3	0.29 (0.06-1.38)			
Wu [26]	**Categories of intake**	**HR (95%-CI)**	**Adjusted for**	**p-trend**	+
	<1 portions/week	1.0 (ref)	age, family history of melanoma, natural hair color, No. of arm moles, sunburn susceptibility as child or adolescent, No. of lifetime blistering sunburns, cumulative ultraviolet flux since baseline, average time spent in direct sunlight since high school, BMI, physical activity, smoking status, intake of total energy, alcohol, coffee, vitamin C supplements, menopausal status and postmenopausal hormone use (only women)	<0.001	
	1-2 portions/week	1.07 (0.93-1.24)			
	3-4 portions/week	1.04 (0.84-1.30)			
	5-6 portions/week	1.22 (1.05-1.42)			
	≥1 portions/day	1.25 (1.07-1.47)			
**Other citrus fruits**					
**Orange, tangerine, tangelo**					
Melough [39]	**Categories of intake**	**HR (95%-CI)**	**Adjusted for**	**p-trend**	o
	None	1.00 (ref)	age, sex, cigarette smoking, BMI, education, alcohol intake, physical activity, family history of cancer, July erythemal exposure	0.38	
	<0.25 cups/week	1.03 (0.92-1.15)			
	0.25 - <0.75 cups/week	1.01 (0.09-1.14)			
	0.75 - <2 cups/week	1.04 (0.92-1.18)			
	2+ cups/week	1.03 (0.91-1.17)			

**Mandarin**					
Marley [38]	**Categories of intake**	**OR (95%-CI)**	**Adjusted for**	**p-trend**	o
	None	1.0 (ref)	age, sex, education, income, physical activity, BMI, smoking status, alcohol intake, coffee intake, tanning ability, childhood sunburn occasions, natural hair color, skin color, average time spent outdoors in summer, sunlamp or solarium use, use of sun/ultraviolet protection, total energy intake, history of NMSC, consumption of other individual citrus products	0.11	
	>0-0.5 portions/day	1.19 (0.98-1.43)			
	>0.5-1 portions/day	1.04 (0.82-1.30)			
	>1 portions/day	1.29 (0.092-1.74)			
**Orange and grapefruit**					
Malavolti [22]	**Tertiles of intake**	**OR (95%-CI)**	**Adjusted for**	**p-trend**	o
	T1	1.0 (ref)	vitamin D intake, tanning habits, and sunburns history	0.363	
	T2	1.02 (0.70-1.47)			
	T3	1.08 (0.76-1.51)			
**Orange and mandarin**					
Grasgruber [34]	Pearson's linear correlations:	r = 0.48 (men); r = 0.52 (women)	No adjustment possible due to design		o
**Orange juice and grapefruit juice**					
Malavolti [22]	**Tertiles of intake**	**OR (95%-CI)**	**Adjusted for**	**p-trend**	o
	T1	1.0 (ref)	vitamin D intake, tanning habits, and sunburns history	0.178	
	T2	1.01 (0.72-1.41)			
	T3	0.82 (0.59-1.12)			
**Tangerine**					
Malavolti [22]	**Tertiles of intake**	**OR (95%-CI)**	**Adjusted for**	**p-trend**	o
	T1	1.0 (ref)	vitamin D intake, tanning habits, and sunburns history	0.935	
	T2	1.12 (0.81-1.57)			
	T3	1.07 (0.78-1.47))			

**Others**					
**Parsely**					
Fortes [32]	**Categories of intake**	**OR (95%-CI)**	**Adjusted for**		o
	No	1.0 (ref)	age, sex, education, hair color, skin phototypes, number of nevi, presence of freckles in childhood and sunburns in childhood		
	Yes	0.94 (0.46-1.95)			
**Carrot**					
Fortes [32]	**Frequency of intake**	**OR (95%-CI)**	**Adjusted for**		-
	Low (less than weekly)	1.0 (ref)	age, sex, education, hair color, skin phototypes, number of nevi, presence of freckles in childhood and sunburns in childhood		
	High (weekly and more)	0.57 (0.38-0.84)			
Holman [20]	No quantitative results: "Examination of food frequency results relating to carrots […] also revealed no evidence of effect."				o
Naldi [30]	**Tertile of intake**	**OR (95%-CI)**	**Adjusted for**	**p-trend**	-
	T1 (0 portions/week)	1.0 (ref)	age, sex, education, BMI, history of sunburns, propensity to sunburn, number of nevi, number of freckles, skin, hair and eye color, and tobacco smoking	0.006	
	T2 (<1 portions/week)	0.98 (0.62-1.53)			
	T3 (≥1 portions/week)	0.66 (0.44-0.99)			
Østerlind [27]	Exposed vs. not exposed	RR = 1.2 (0.6-2.2)	No adjustment	p = 0.09	o
Soliman [21]	**Level of intake**	**OR**	**Adjusted for**	**p-trend**	o
	Low	1.0	age, tanning, number of palpable moles, total calories, sun exposure, year of recruitment into the study, gender	0.9	
	Moderate	1.0			
	High	1.0			
Stryker [28]	**Level of intake**	**OR**	**Adjusted for**	**p-trend**	o
	Low	1.0 (ref)	age, sex, hair color, ability to tan	0.8	
	Moderate	1.1			
	High	1.1			

Total furocoumarin consumption					
Melough [36]	**Categories of intake**	**OR (95%-CI)**	**Adjusted for**		+
	Non- or Low Consumers (<50th %ile)	1.00 (ref)	age, gender, total energy intake, ethnicity, poverty income, ratio, smoking, and physical activity		
	Moderate Consumers (50th to 80th %iles)	0.90 (0.45-1.78)			
	High Consumers (80th to 90th %iles)	1.66 (0.39-7.16)			
	Very High Consumers (>90th %iles)	1.75 (0.43-7.20)			
Sun [25]	**Quintiles of intake**	**HR (95%-CI)**	**Adjusted for**	**p-trend**	+
	Q1	1.00 (ref)	age, calendar time, No. of lifetime blistering sunburns, No. of arm moles, natural hair color, sunburn susceptibility as child or adolescent, family history of melanoma, alcohol intake, UV exposure at residence, physical activity, routine physical examination, intake of total energy, history of basal cell carcinoma and squamous cell carcinoma	0.64	
	Q2	1.15 (0.98-1.36)			
	Q3	1.21 (1.02-1.42)			
	Q4	1.24 (1.06-1.46)			
	Q5	1.11 (0.94-1.31)			

HR: hazard ration, OR: odds ratio, RR: relative risk, BMI: body mass index, NMSC: non-melanoma skin cancer, UV: ultraviolet

+: positive association, o: no association, -: negative association.
